# Selection of Resistant Bacteria at Very Low Antibiotic Concentrations

**DOI:** 10.1371/journal.ppat.1002158

**Published:** 2011-07-21

**Authors:** Erik Gullberg, Sha Cao, Otto G. Berg, Carolina Ilbäck, Linus Sandegren, Diarmaid Hughes, Dan I. Andersson

**Affiliations:** 1 Department of Medical Biochemistry and Microbiology, Uppsala University, Uppsala, Sweden; 2 Department of Molecular Evolution, Uppsala University, Uppsala, Sweden; Harvard School of Public Health, United States of America

## Abstract

The widespread use of antibiotics is selecting for a variety of resistance mechanisms that seriously challenge our ability to treat bacterial infections. Resistant bacteria can be selected at the high concentrations of antibiotics used therapeutically, but what role the much lower antibiotic concentrations present in many environments plays in selection remains largely unclear. Here we show using highly sensitive competition experiments that selection of resistant bacteria occurs at extremely low antibiotic concentrations. Thus, for three clinically important antibiotics, drug concentrations up to several hundred-fold below the minimal inhibitory concentration of susceptible bacteria could enrich for resistant bacteria, even when present at a very low initial fraction. We also show that *de novo* mutants can be selected at sub-MIC concentrations of antibiotics, and we provide a mathematical model predicting how rapidly such mutants would take over in a susceptible population. These results add another dimension to the evolution of resistance and suggest that the low antibiotic concentrations found in many natural environments are important for enrichment and maintenance of resistance in bacterial populations.

## Author Summary

Antibiotic resistance has emerged as a very significant health care problem due to the extensive use and misuse of antibiotics in human and veterinary medicine and in agriculture. It remains unclear where most of the resistant bacteria have been selected, and in particular if the low antibiotic concentrations that are present in natural environments or in human/animal body compartments during therapeutic or growth promotion use, are important for the selection and enrichment of resistant mutants. The presented data shows that for several clinically used antibiotics extremely low concentrations, similar to the concentrations found in natural environments, can select for resistant bacteria. These results suggest that antibiotic release into the environment might be a significant contributor to the emergence and maintenance of resistance and emphasize the importance of introducing measures to reduce antibiotic pollution.

## Introduction

**Figure 1:**
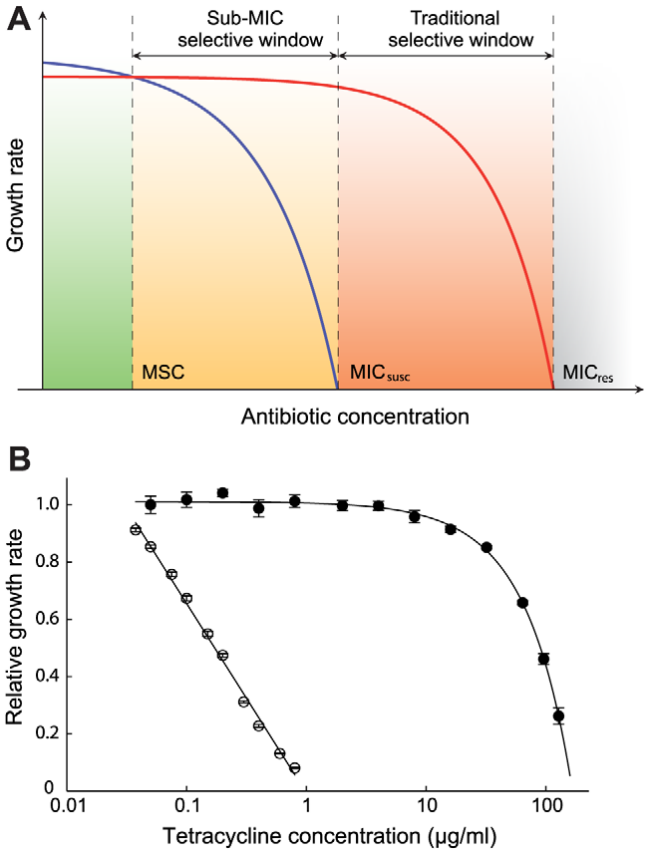
Growth rates as a function of antibiotic concentration. (**A**) Schematic representation of growth rates as a function of antibiotic concentration. Green indicates a concentration interval where the susceptible strain (blue line) will outcompete the resistant strain (red line). Orange (sub-MIC selective window) and red (traditional mutant selective window) indicate concentration intervals where the resistant strain will outcompete the susceptible strain. MIC_susc_ =  minimal inhibitory concentration of the susceptible strain, MIC_res_ =  minimal inhibitory concentration of the resistant strain and MSC =  minimal selective concentration. (**B**). Relative exponential growth rates of susceptible (open circles) and resistant (closed circles) strains of *S. typhimurium* as a function of tetracycline concentration. Standard errors of the mean are indicated. A relative growth rate of 1.0 corresponds to approximately 1.8 hr^−1^. Cells were grown in Mueller Hinton medium at 37°C.

Antibiotics represent one of mankind's most important medical inventions but during the last decades the continuing rapid development of antibiotic resistance has emerged as one of the most serious health care problems, both in community and hospital settings [1], [2], [3]. Whereas some resistance-conferring genes were most likely originally selected to serve metabolic functions and/or for signal trafficking or protection against competing antibiotic-producing bacteria [4], the recent worldwide enrichment and spread of highly resistant pathogenic bacteria in the micro-biosphere has largely been driven by human activities, including the extensive use and misuse of antibiotics in human and veterinary medicine and in agriculture [2], [3], [5], [6], [7]. While it is evident that the high concentrations of antibiotics used therapeutically can select for resistant mutants, it still remains unclear how important the low antibiotic concentrations that due to anthropogenic input pollute natural (e.g. aquatic or soil) environments [8], [9], [10], that are produced naturally by antibiotic-producing micro-organisms or that are present in certain human/animal body compartments during therapeutic or growth promotion use, are for the selection and enrichment of resistant mutants. In pharmacodynamic models it is generally assumed that selection of resistant bacteria only occurs at concentrations between the minimal inhibitory concentration (MIC) of the susceptible wild type population (MIC_susc_) and that of the resistant bacteria (MIC_res_) [11], [12] (mutant selective window hypothesis, see Fig. 1A) and that concentrations below the MIC_susc_ will not inhibit growth of the susceptible bacteria and therefore not be selective. Earlier studies on selection with small differences in bacterial susceptibility to antibiotics show that selection can efficiently act on minute differences to select for resistance [13], [14], [15]. Furthermore, using an elegant color-based assay a recent study has shown qualitatively that levels of antibiotics below the MIC can enrich for resistant bacteria [16]. Here, we further explore the mutant selective window assumption and as outlined schematically in Fig. 1A, we examine for two bacterial species and three antibiotics how far below the MIC_susc_ pre-existing and *de novo* generated resistant mutants can be selectively enriched because of minute reductions in the growth rate of their susceptible counterparts.

To determine if exposure to very low antibiotic concentrations (<<MIC_susc_) can result in enrichment for resistant mutants, we used several well-defined mutants of *Escherichia coli* and *Salmonella enterica* (Var. Typhimurium LT2) (Table S1 in Text S1) and three different classes of antibiotics with high importance to human and veterinary medicine (tetracyclines, fluoroquinolones and aminoglycosides). The resistance markers used were Tn*10d*Tet (confers tetracycline resistance), *gyrA* (S83L and D87N), Δ*marR,* and *ΔacrR* mutations (confer ciprofloxacin resistance) and *rpsL* (K42R) (confers streptomycin resistance), all of which are found in clinical isolates of several different bacterial species. Using highly sensitive competition experiments between isogenic pairs of susceptible and resistant strains, we show that selection of resistant bacteria can occur at antibiotic concentrations far below the minimal inhibitory concentration. Finally, we present a mathematical model, showing how resistant mutants are expected to arise *de novo* and spread in bacterial populations at these sub-MIC levels of antibiotics.

## Results

**Figure 2:**
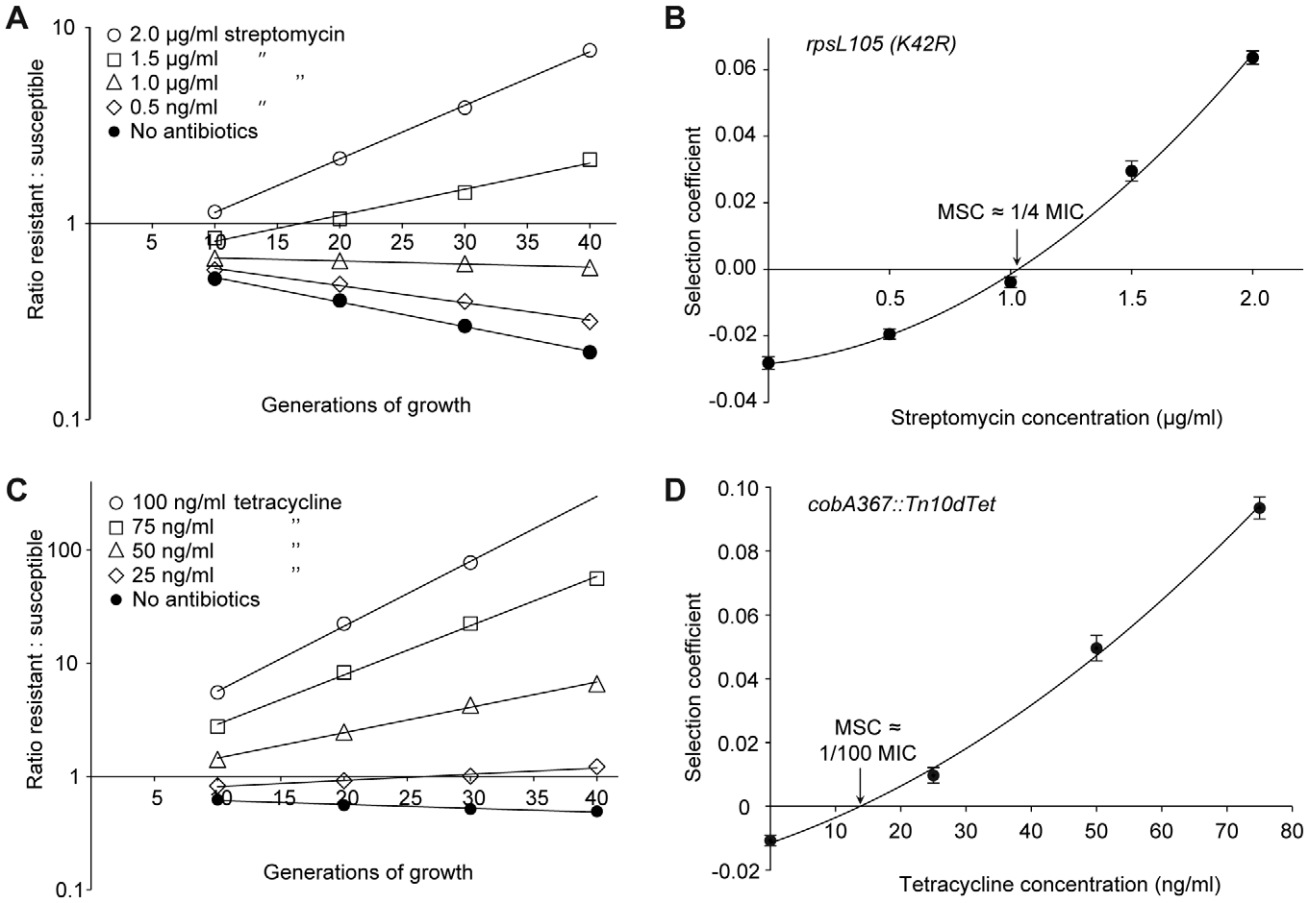
Competition experiments between susceptible and resistant strains, streptomycin and tetracycline. Competition experiments at different concentrations of antibiotics (A (*rpsL105 (K42R)*) and C (*Tn*10*d*tet), and calculated selection coefficients as a function of antibiotic concentrations (B and D). Fig. A and C are each based on one single competition experiment (averages of four competitions), while Fig. B and D are calculated from the selection coefficients of up to 20 competitions (Table S3 in Text S1). Standard errors of the mean are indicated.

**Figure 3:**
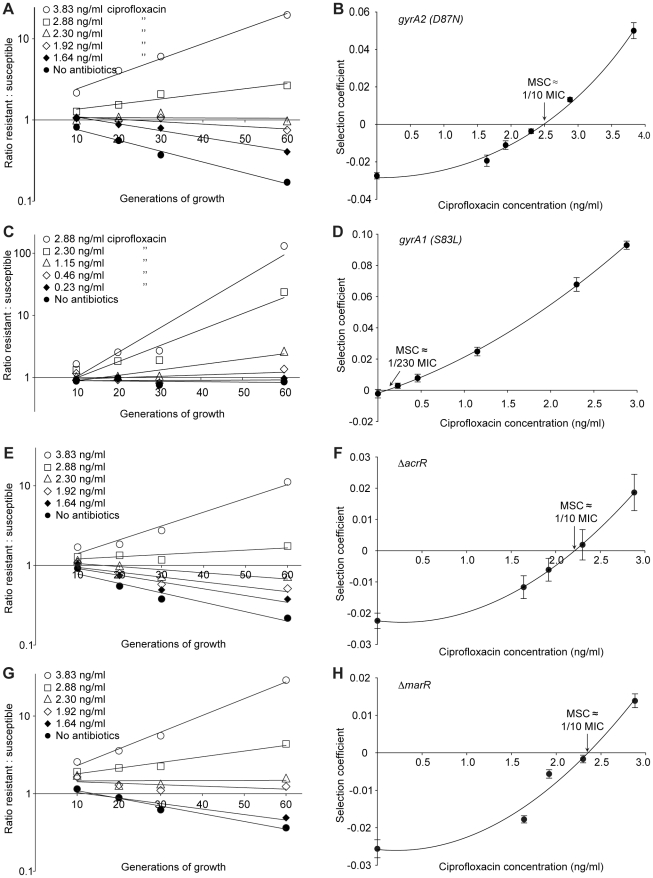
Competition experiments between susceptible and resistant strains, ciprofloxacin. Competition experiments at different concentrations of ciprofloxacin (A (gyrA2 (D87N)), C (gyrA1 (S83L)), E (*ΔacrR*) and G (*ΔmarR*)) and calculated selection coefficients as a function of antibiotic concentrations (B, D, F and H). Fig. A, C, E and G are each based on one single competition experiment (averages of three competitions), while fig. B, D, F and H are calculated from the selection coefficients of 6 competitions (Table S3 in Text S1). Standard errors of the mean are indicated.

**Figure 4:**
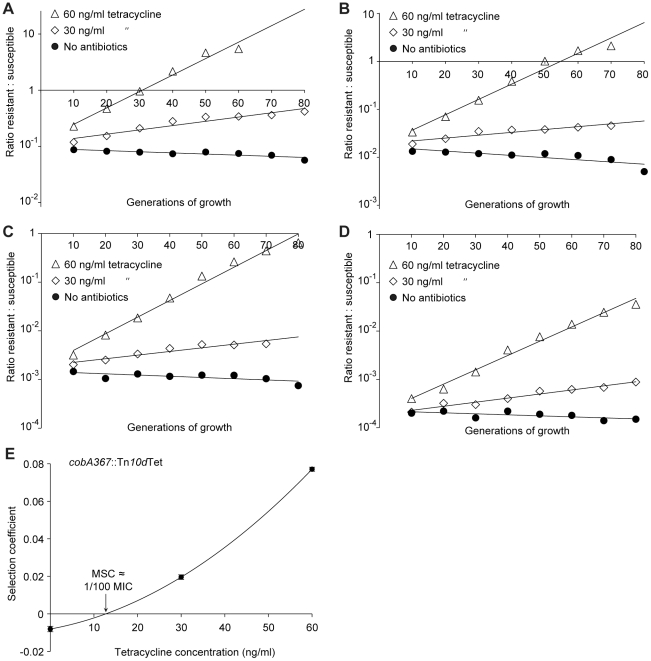
Competition experiments with low initial frequencies of resistant mutants. Competition experiments at different concentrations of antibiotics and different starting fractions of resistant mutants. (A) Initial ratio of susceptible to resistant mutants 10∶1. (B) Initial ratio of susceptible to resistant mutants 10^2^∶1. (C) Initial ratio of susceptible to resistant mutants 10^3^∶1. (D) Initial ratio of susceptible to resistant mutants 10^4^∶1. (E) Calculated selection coefficients as a function of antibiotic concentrations. Fig. A to D are each based on one single competition experiment while E is calculated from the selection coefficients of 24 independent competitions with four different starting fractions of resistant mutants (Table S3 in Text S1). Standard errors of the mean are indicated.

An initial examination of the effect of low antibiotic concentrations was performed in single cultures where a susceptible wild-type and a resistant mutant carrying a Tn*10d*Tet were grown separately in the presence of different concentrations of tetracycline. As shown in Fig. 1B and Table S2 in Text S1, concentrations far below MIC_susc_ reduced the exponential growth rate of the susceptible strain without any apparent effect on the resistant strain. For example, at a concentration 1/30 of the MIC_susc_, the susceptible strain grew about 15% slower than without antibiotic whereas the resistant mutant seemed unaffected, suggesting that resistant strains are strongly selected at these low concentrations. To increase the sensitivity of these assays and allow detection of extremely small differences in growth rates, we performed competition experiments between pairs of susceptible and resistant strains. The MICs for the susceptible and resistant mutants were: *S. typhimurium* wild type (streptomycin = 4 ug/ml, tetracycline = 1.5 ug/ml), *rpsL K42R* >1024 ug/ml and Tn*10d*Tet strain  = 128 ug/ml; *E. coli* wild type (ciprofloxacin = 0.023 ug/ml), *gyrA* S83L (ciprofloxacin = 0.38 ug/ml), *gyrA* D87N (ciprofloxacin = 0.25 ug/ml), Δ*acrR* (ciprofloxacin = 0.047 ug/ml) and Δ*marR* (ciprofloxacin = 0.047 ug/ml). The strains were genetically tagged with variants of the green fluorescent protein gene (*yfp* and *cfp,* encoding yellow- and cyan-fluorescent proteins, respectively) to allow counting of large populations of competing cells by fluorescence activated cell sorting (FACS), thereby significantly reducing any experimental errors associated with counting of small populations. The competing strains were isogenic except for the resistance determinant and the *yfp* and *cfp* genes producing the respective fluorescent proteins. Control experiments showed that the difference in fitness cost between the *cfp* and *yfp* markers had a negligible impact on growth rates (Fig. S1). The strains were competed for up to 80 generations by serial passage in batch cultures in the presence of different concentrations of either one of the antibiotics tetracycline, ciprofloxacin (a fluoroquinolone) and streptomycin (an aminoglycoside) as well as in the absence of drug (Fig. 2A–D, Fig. 3A–H, Fig. 4A–E). As shown by our previous studies [17], this experimental set-up allows detection of growth rate differences at least as small as 0.3%, which approaches the limit of sensitivity set by the interference of periodic selection events. Whereas the growth rate measurement shown in Fig. 1B only measured the exponential phase of growth, the competition experiments represent a composite of growth and survival in lag phase, exponential phase and stationary phase that allows examination of the whole growth cycle.

The data presented in Fig. 2A, C, Fig. 3A, C, E, G and Fig. 4A–D shows how the ratio of resistant:susceptible strains changes as a function of the number of generations of growth at different concentrations of antibiotic. Each line represents one competition experiment and the slope is a measure of the selection coefficient (s-value). Thus, the negative slope obtained in the absence of antibiotic is a measure of the fitness cost of the antibiotic resistance mechanism and a positive slope indicates that the resistant mutant is enriched. When the s-values obtained from these experiments are plotted as a function of antibiotic concentration the intercept, s = 0, represents what we specify as the minimal selective concentration (MSC) where the fitness cost of the resistance is balanced by the antibiotic-conferred selection for the resistant mutant (Fig. 2B, D, Fig. 3B, D, F, H and Fig. 4E). Depending on the antibiotic and the type of resistance mutation examined the MSC varied between 1/4 and 1/230 of the MIC_susc_. For streptomycin the MSC value was 1/4 of the MIC value of the susceptible strain (Fig. 2B), for tetracycline 1/100 (Fig. 2D) and for ciprofloxacin it varied between 1/10 (Fig. 3B) and 1/230 (Fig. 3D) of the MIC_susc_ depending on the particular resistance mutation. These values correspond to absolute antibiotic concentrations of 1 µg/ml (streptomycin), 15 ng/ml (tetracycline), and 2.5 ng/ml to 100 pg/ml (ciprofloxacin). The competitions performed with a small initial fraction of resistant mutants also showed that the selection coefficients are independent of the initial frequency of resistant mutants. Even at initial frequencies as low as 10^−4^, the same enrichment (i.e. same selection coefficient) of the resistant mutants could be observed as at a 1∶1 ratio (compare Figures 2D and 4E).

**Figure 5:**
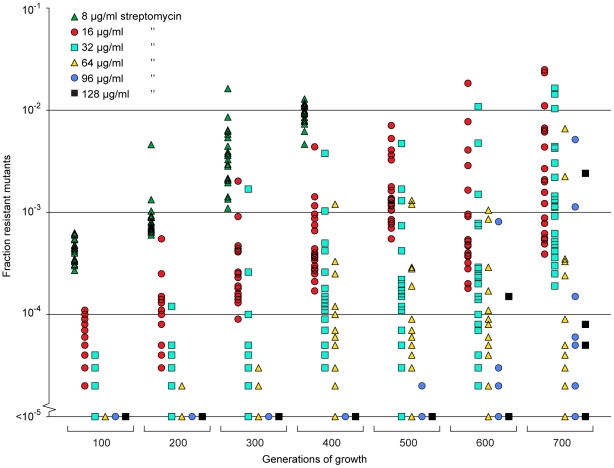
Selection of *de novo* resistant mutants at sub-inhibitory concentrations of antibiotics. A total of 20 independent lineages of *S. typhimurium* were serially passaged in Mueller-Hinton medium containing 1 µg/ml streptomycin. Every 100 generations approximately 10^5^ cells were plated onto LB agar containing different concentrations of streptomycin and the fractions of resistant mutants were calculated. The data points are grouped by number of generations of growth and resistance level, and in each of these data sets one data point represents the fraction of cells present in one lineage capable of growth at the specified antibiotic concentrations. Please note that data points at the baseline will overlap.

Since the resistant mutants could be enriched from very low initial fractions (10^−4^) we also tested whether resistant mutants could be selected *de novo* from a susceptible population. To this end we grew 20 independent lineages of a susceptible wild type *Salmonella typhimurium* LT2 strain for 700 generations at 1/4 of the MIC of streptomycin and continuously screened for resistant cells by plating on different concentrations of streptomycin. At this low level of antibiotic we could observe rapid enrichment of *de novo* resistant mutants (Fig. 5). Thus, within 200 to 400 generations, a considerable enrichment of mutants with resistances between 2 and 16 times the MIC of the starting strain (8–64 µg/ml) could be seen, and after 500 to 600 generations also high-level resistant mutants (24–32 times MIC of the wild type  = 96–128 µg/ml) appeared. After 400 generations, all 20 lineages contained subpopulations with a MIC higher than 32 µg/ml (8 times MIC), and after 600 generations 14 of the lineages had subpopulations with a MIC higher than 64 µg/ml (16 times MIC). Using the method described above, 20 lineages of wild type *E. coli* were grown for 600 generations in sub-MIC levels of ciprofloxacin. After 500 generations of growth at 1/10 of the MIC, five of the lineages had subpopulations (>1% of the population) with low level resistance (2-fold higher MIC than the susceptible parental strain) to ciprofloxacin, and after 600 generations, one out of twenty lineages had a subpopulation of cells with an MIC 8-fold higher than the susceptible parental strain (see Fig. S2).

We also calculated (Appendix in Text S1) how rapidly *de novo* generated resistant mutants would take over in a susceptible population at low antibiotic concentration, as determined by mutation rates (*u*), population sizes (*N*), and the fitness advantage (*s*) in the presence of antibiotics. *s* depends on the antibiotic concentration above the MSC as shown in Fig. 2 and 3. When no resistance mutants are present initially, the time to fixation can be expressed as
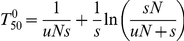



**Figure 6:**
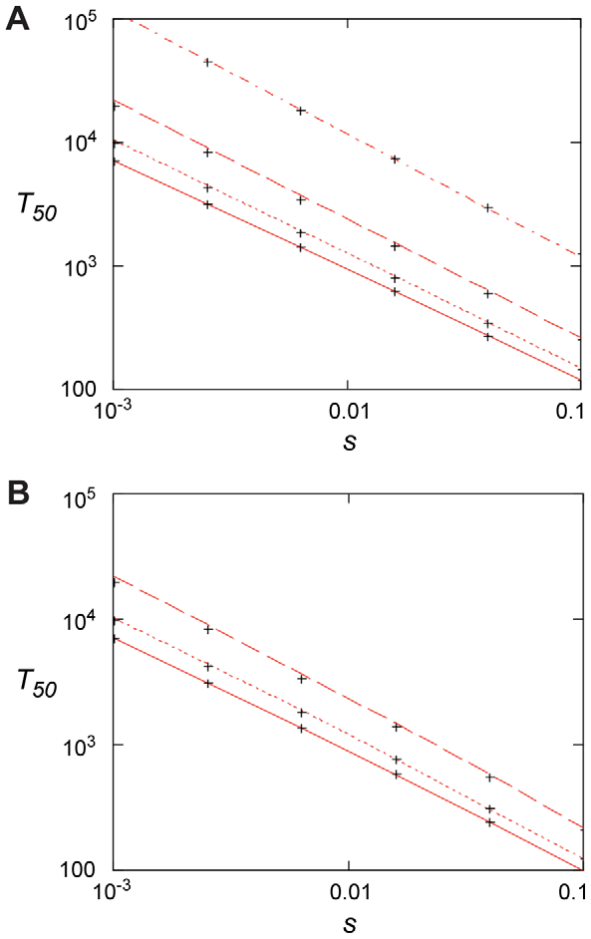
Fixation time for adaptive mutations. (A) 50% penetration time as function of selection coefficient when no mutants are present initially for *N* = 10^7^ and, from top to bottom, *u* = 10^−9^, 10^−8^, 10^−7^, and 10^−6^. Red lines are from Eq. (4) and the crosses from the stochastic model, Eqs. (7) – (10), with *m*_0_  = 0. (B). 50% penetration time as function of the selection coefficient when the initial presence of mutants is determined by mutation-selection balance, *m*_0_  =  *Nf*_0_  =  *uN*/|*s*_0_|. Results are for *s*_0_  =  −0.02, *N* = 10^7^ and, from top to bottom, *u* = 10^−6^, 10^−7^, 10^−8^. The red lines are from Eq. (6), and the crosses are from the stochastic model, Eqs. (7) – (10).

The first term is the stochastic waiting time for the first surviving mutant to appear and the second term is from the subsequent growth to 50% presence. For small values of *uN* <0.1, the first term dominates and fixation may be slow. For large values, *uN* >1, the second term dominates and fixation can be fast, ca. 100–1000 generations for *s* between 0.1–0.01 (Fig. 6). In this limit, resistance mutants appear so frequently that it makes little difference to the fixation time if they are present initially or not. In this context it is worth noting that sub-MIC levels of several antibiotics, most pronounced for fluoroquinolones, have been shown to increase bacterial mutation rates which potentially could reduce the waiting time and thereby increase the rate of mutant take-over [18].

## Discussion

Antibiotic concentrations in natural environments can vary extensively depending on the particular environment. For example, in connection with polluting pharmaceutical industries or at sewage outlets from hospitals the concentrations can reach very high levels (mg/ml), with fluoroquinolones frequently reaching the highest levels [19], [20], [21] whereas in aquatic environments or in soil levels are typically much lower [8]. The presented data suggests that even in those environments with very low antibiotic concentrations, maintenance and selection of resistant bacteria can occur. For example, the MSC for ciprofloxacin and tetracycline obtained from our experiments correspond to 100 pg/ml and 15 ng/ml, respectively, similar to concentrations that can be found in some aquatic and soil environments [8]. Thus, the surprisingly high frequencies of antibiotic-resistant bacteria found in animals from relatively pristine environments [22], [23], [24] could conceivably be partly explained by enrichment due to sub-MIC selective effects.

These findings are also highly relevant with regard to the question of reversibility of resistance. Since most antibiotic resistance mechanisms are associated with a fitness cost it has been proposed that the fitness costs of resistance will allow susceptible bacteria to out-compete resistant bacteria if the antibiotic selective pressure is reduced. However, most available data suggests that the rate of reversibility will be slow or absent at the community level [25]. Several factors could contribute to this irreversibility, including the absence of a fitness cost, reduction of the fitness cost through compensating mutations and genetic co-selection between the resistance-conferring gene and another gene under selection. In addition, the sub-MIC selection observed here could be a significant contributor to this long-term persistence of resistance where very low antibiotic concentrations in the environment are sufficient to maintain the existing resistant bacteria in the population by further balancing the fitness cost of the resistance. This can be particularly important for bacterial pathogens whose normal life cycle involves growth in soil environments (e.g. *P. aeruginosa*) or periodic growth in aquatic environments (e.g. *E. coli*).

From the slope of the graphs in Fig. 2B, D and Fig. 3B, D, F and H we can infer that the fitness cost of the resistance mutation has a major influence on the value of the MSC. This cost must first be overcome by a negative effect of antibiotics on the susceptible bacteria before resistant bacteria will be selected, shifting the MSC towards higher concentrations. Reducing this cost will shift the curve upwards and lower the MSC. It is also evident that the increased resistance of the mutants (difference between MIC_susc._ and MIC_res_.) or the mode of resistance (point mutation or efflux pumps) has little effect on MSC relative to the fitness cost. Since our data was obtained in defined genetic backgrounds with single point mutations or deletions commonly found for the antibiotics tested, the fitness cost represents the cost of a *de novo* resistance mutation. However, in most resistant strains found clinically the fitness cost of resistance is frequently compensated for by secondary mutations without a loss of resistance [25]. Such fitness compensation has been described for resistance to many different antibiotics including fluoroquinolones and streptomycin [26], [27], [28]. This implies that the antibiotic concentrations at which such compensated resistant strains will be selected can be even lower than what we have measured here.

Another significant implication from our and the findings of others is that the widely used concept of the mutant selective window needs modification. Thus, in pharmacodynamics it is generally assumed that antibiotic concentrations below the MIC do not confer selection and that the mutant selective window—the concentration range in which the resistant mutant is enriched—extends between the MIC of the susceptible wild type and the MIC of the resistant mutants [11], [12]. However, our results imply that the biologically relevant sub-MIC selective window is much wider and needs to include antibiotic concentrations several hundred-fold below MIC_susc_ (Fig. 1A). Furthermore, the methodology described here could be used to probe the biologically active antibiotic concentrations in different environments, including for example animal models. Thus, by performing competitions between genetically tagged susceptible and resistant strains in animals treated with different antibiotic concentrations one can from the enrichment rate of resistant bacteria infer the biologically active concentration of antibiotic at the site of bacterial growth.

At selection above the MIC of a strain, the main driving force of the selection is antibiotic resistance, while the fitness cost of the mutation is less critical. Even mutations with a very high cost will be selected, since competitors in the form of susceptible bacteria will be eliminated. At sub-MIC levels, however, the situation is different since the susceptible bacteria will not die, they will only grow slower. Because of this, resistance mutations conferring high fitness costs will not be enriched; only mutations where the fitness cost is lower than the growth reduction caused by the antibiotic in the susceptible bacteria will be competitive. This suggests that a new spectrum of low-cost or no-cost resistance mutations might be enriched during such conditions. The data in Fig. 5, show that these sub-MIC levels of antibiotics do not only enrich for pre-existing resistant mutants, but they can also select for resistant mutants *de novo* from a susceptible population. It is interesting to note that despite the low antibiotic concentrations used, mutants with high resistance levels were enriched. Since the streptomycin concentration chosen for the *de novo* mutant selection experiment is the same as the MSC determined in the competitions between wild type and an *rpsL K42R* mutant, the enriched resistant bacteria are likely to carry resistance mutations with a fitness cost that is significantly lower than the previously studied *rpsL* mutation.

In the presented experiments pre-existing mutants were rapidly enriched in competitions with susceptible strains. From the mathematical model we can infer a similar situation for *de novo* resistant mutants, especially in large populations where *uN* >1 and at antibiotic concentrations where 0.01< s <1.0. In those situations resistant mutants rapidly appear and within 100–1000 generations of growth they will take over the population. The model is supported by the experiments shown in Fig. 5 and Fig. S2, where *de novo* mutants continuously increased in frequency during 600–700 generations of growth in the presence of sub-MIC levels of antibiotics.

In conclusion, the presented data suggests that the very low antibiotic levels which are present in many natural environments or generated in certain body compartments during treatment are relevant for the enrichment and maintenance of pre-existing resistant mutants as well as for the *de novo* selection of new mutants. These results emphasize the importance of introducing measures that reduce antibiotic levels in the environment and use of treatment dosing regimens that preclude prolonged time periods of sub-MIC levels of antibiotics.

## Materials and Methods

### Bacterial strains, genetic methods, and growth conditions

Strains used in this study were derived from *Escherichia coli* MG1655 and *Salmonella enterica* serovar Typhimurium LT2 (designated *S. typhimurium* in the text) and are listed in Table S1 in Text S1. The resistant strains were constructed by P22 transduction (*S typhimurium*) or P1 transduction (*E coli*) of the resistance genes into the parental strains. The liquid and solid media used for bacterial growth were Mueller–Hinton broth (Becton Dickinson, MD, USA), Mueller–Hinton agar (Mueller–Hinton broth supplemented with 1.5% agar) and Luria–Bertani (LB) agar (Sigma-Aldrich, MO, USA). Strains were grown at 37°C, and liquid cultures were aerated by shaking.

### Growth rate measurements

Growth rates were measured at 37°C in Mueller-Hinton broth, with or without tetracycline present, using a Bioscreen C Analyzer (Oy Growth Curves Ab Ltd, Helsinki, Finland). Each well was inoculated with a 1000-fold dilution of an overnight culture and measurements at each antibiotic concentration were made in quadruplicate. The cultures were grown for 24 hours with continuous shaking, and OD_600_ measurements were taken every 4 min. The calculations were based on OD_600_ values between 0.02 and 0.1, where growth was observed to be exponential. The sensitive strain (DA6192) and the resistant strain (DA17822) were grown in separate experiments, and the relative growth rates were calculated as the derived growth rates divided by the growth rate of the same strain grown without antibiotics.

### MIC measurements

MIC assays of tetracycline and ciprofloxacin were performed by broth macrodilution in 10 mL tubes. Tubes containing Mueller-Hinton broth (1 mL) supplemented with different concentrations of antibiotics were inoculated with 1 µL of an overnight bacterial culture grown at 37°C. The tubes were incubated at 37°C with shaking for 16 to 18 hours, the tetracycline cultures protected from light to avoid degradation of the antibiotic. The MIC was set to the lowest concentration of antibiotic yielding no visible growth. The MIC of streptomycin was determined by Etest according to the instructions of the manufacturer (AB bioMerieux, Solna, Sweden). Etests were performed on Mueller-Hinton agar plates incubated for 16–18 h at 37°C.

### Competition experiments

Limited sampling of competitors (<10^3^ cells) commonly introduces statistical uncertainties in competition experiments and more accurate measurements of resistant mutant to wild type cell ratios can be obtained with the aid of chromosomal copies of either the cyan (*cfp*) or yellow (*yfp*) variants of green fluorescent protein gene (*gfp*). These allow tracking of large numbers of single cells (10^5^ cells) using a fluorescence activated cell sorter (FACS). The *cfp*/*yfp* genes were inserted into *galK* using the λ Red system as previously described [29] and moved by phage P22 transduction or phage P1 transduction into the various strains.

Overnight cultures grown in Mueller-Hinton medium of the susceptible wild type strains with either *cfp* or *yfp,* were mixed 1∶1, 10∶1, 10^2^∶1, 10^3^∶1 and 10^4^∶1 with the isogenic resistant mutant carrying the other marker and maintained by 1000-fold serial dilution (resulting in 10 generations of growth per serial passage) every 24 hours for up to 4 to 6 serial passages. The ratio of resistant to susceptible cells in the population was determined at each serial passage by counting 10^5^ cells using a fluorescence-activated cell sorter (BD FacsAria). The selection coefficients were determined using the regression model s  =  [ln(R(t)/R(0))]/[t], as previously described [30] where R is the ratio of resistant to susceptible. This protocol allowed reproducible determinations of fitness differences as small as s  = 0.003 [17]. Two independently constructed sets of each wild type strain, marked with either *cfp* or *yfp,* were also included to measure the relative impact on growth rates of having a *cfp* marker compared to *yfp.* These control experiments showed that over 40 generations of competition, the difference in cost between the markers had a negligible impact on growth rates. (Fig. S1). The competition experiments performed with a low initial fraction of resistant mutants were done with tetracycline due to the long time required for the appearance of *de novo* tetracycline resistant mutants that might disturb the competition experiments.

### Enrichment of *de novo* evolved resistant mutants

To investigate whether sub-inhibitory antibiotic concentration could also select for *de novo* generated resistant mutants, susceptible bacteria was serially passaged at 1/4 of the MIC of streptomycin and at 1/10 of the MIC of ciprofloxacin. A total of 20 independent lineages of *S. typhimurium* LT2 was serially passaged by 1000-fold dilution in 1 ml batch cultures every 24 hours for 700 generations (10 generations of growth per serial passage) in Mueller-Hinton medium containing 1 µg/ml streptomycin, and 20 independent lineages of *E. coli* MG1655 were serially passaged by 1000-fold dilution in 1 ml batch cultures every 24 hours for 600 generations in Mueller-Hinton medium containing 2.3 ng/ml ciprofloxacin. The lineages were started from overnight cultures from independent colonies, using an initial bottleneck of approximately 10^4^ cells to minimize the number of preexisting resistant mutants. The percentage of resistant cells in each culture was monitored by plating approximately 10^5^ cells onto LB agar containing different concentrations of antibiotics every 100 generations and counting the number of colonies. A subset of these cells were restreaked on the same antibiotic concentration to confirm that they were resistant.

### Accession numbers

Tn10 (Transposon Tn10 tetracycline resistance and repressor genes *tetA* and *tetR*)

GenBank: J01830.1

*rpsL* (30S ribosomal protein S12)

GenBank: AAL22311.1

Swiss-Prot: P0A7S6

*gyrA* (DNA gyrase subunit A)

GenBank: AAC75291.1

Swiss-Prot: P0AES4

*acrR* (HTH-type transcriptional regulator AcrR)

GenBank: AAC73566.1

Swiss-Prot: P0ACS9

*marR* (Multiple antibiotic resistance protein MarR)

GenBank: AAC74603.2

Swiss-Prot: P27245

*gfp* (Green fluorescent protein)

GenBank: AAA27722.1

Swiss-Prot: P42212

## Supporting Information

Figure S1 Competition experiments between two wild type *S typhimurium* strains marked with either *yfp* or *cfp* (strains DA15110 and DA15111, Table S1 in Text S1). Each line represents one experiment (averages of four competitions), and a total of four independent experiments were conducted.
(TIF)

Figure S2 Selection of *de novo* resistant mutants at sub-inhibitory concentrations of antibiotics. A total of 20 independent lineages of *E. coli* MG1655 were serially passaged in Mueller-Hinton medium containing 2.3 ng/ml ciprofloxacin. Every 100 generations approximately 10^5^ cells were plated onto LB agar containing different concentrations of ciprofloxacin and the fractions of resistant mutants were calculated. The data points are grouped by number of generations of growth and resistance level, and in each of these data sets one data point represents the fraction of cells present in one lineage capable of growth at the specified antibiotic concentrations. Please note that data points at the baseline will overlap.
(TIF)

Text S1 Contains Table S1 (genotypes and MICs of strains), Table S2 (exponential growth rate data), Table S3 (competition data) and Appendix (fixation time for an adaptive mutation).
(DOC)
