# Supplementary figures and images for: Selection of Resistant Bacteria at Very Low Antibiotic Concentrations
Source: PLoS Pathog. 2011 Jul 21;7(7):e1002158. doi: 10.1371/journal.ppat.1002158 (PMC3141051; doi:10.1371/journal.ppat.1002158)

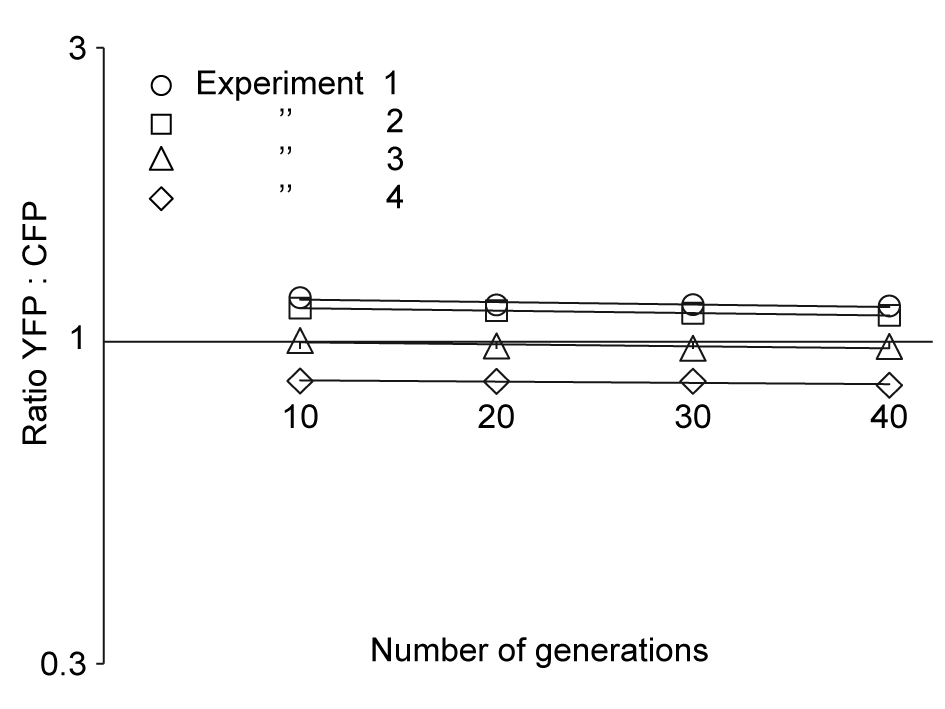

Supplement: Figure S1 — Competition experiments between two wild type S typhimurium strains marked with either yfp or cfp (strains DA15110 and DA15111, Table S1 in Text S1). Each line represents one experiment (averages of four competitions), and a total of four independent experiments were conducted. (TIF) [file ppat.1002158.s001.tif]

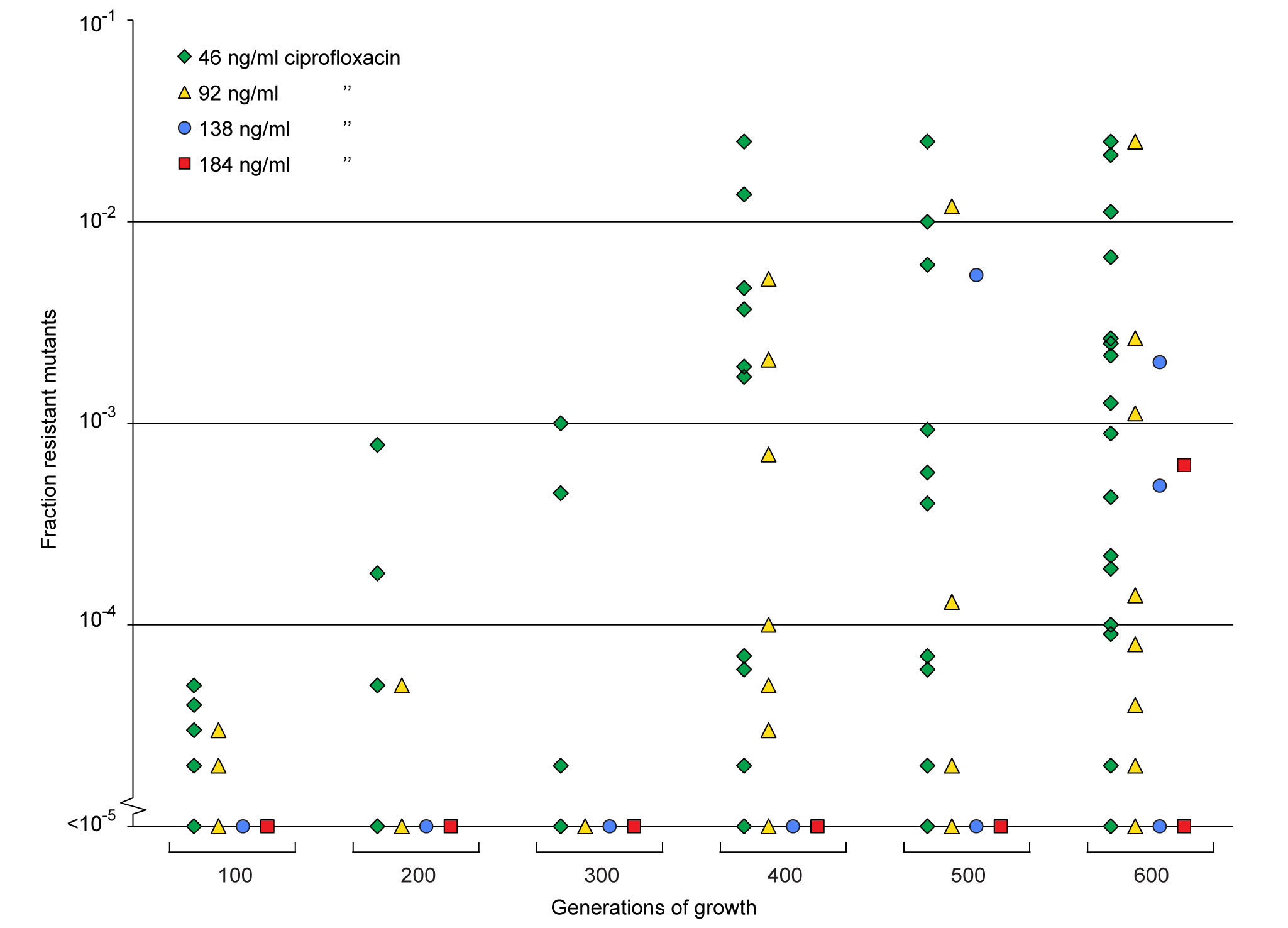

Supplement: Figure S2 — Selection of de novo resistant mutants at sub-inhibitory concentrations of antibiotics. A total of 20 independent lineages of E. coli MG1655 were serially passaged in Mueller-Hinton medium containing 2.3 ng/ml ciprofloxacin. Every 100 generations approximately 105 cells were plated onto LB agar containing different concentrations of ciprofloxacin and the fractions of resistant mutants were calculated. The data points are grouped by number of generations of growth and resistance level, and in each of these data sets one data point represents the fraction of cells present in one lineage capable of growth at the specified antibiotic concentrations. Please note that data points at the baseline will overlap. (TIF) [file ppat.1002158.s002.tif]
