# Supplementary material for: Selection of Resistant Bacteria at Very Low Antibiotic Concentrations
Source: PLoS Pathog. 2011 Jul 21;7(7):e1002158. doi: 10.1371/journal.ppat.1002158 (PMC3141051; doi:10.1371/journal.ppat.1002158)
Supplement: Text S1 — Contains Table S1 (genotypes and MICs of strains), Table S2 (exponential growth rate data), Table S3 (competition data) and Appendix (fixation time for an adaptive mutation). (DOC) [file ppat.1002158.s003.doc]

**Supporting text S1**

**Table S1. Genotypes and MICs of strains.**

| ***Salmonella enterica* serovar Typhimurium strains** | |  | |  |  |
| --- | --- | --- | --- | --- | --- |
|  |  | MIC | | |  |
| Strain | Genotype | Tetracycline (µg/ml) | Streptomycin (µg/ml) | | Reference |
| DA6192 | Wild type LT2 (parent) | 1.5 | 4 | | Strain collection |
| DA15110 | *galK::yfp-bla* | 1.5 | 4 | | Strain collection |
| DA15111 | *galK::cfp-bla* | 1.5 | 4 | | Strain collection |
| DA15966 | *galK::yfp-bla, rpsL105 (K42R)* |  | >1024 | | This study |
| DA17822 | *galK::yfp-bla, cobA367::Tn*10*d*Tet | 128 |  | | This study |
|  |  |  |  | |  |
|  |  |  |  | |  |
| ***Escherichia coli* strains** | |  |  | |  |
| Strain | Genotype | MIC ciprofloxacin (µg/ml) |  | | Reference |
| LM179 | Wild type MG1655 (parent) | 0.023 |  | | Strain collection |
| CH367 | ∆*lac::FRT galK::cfp-bla* | 0.023 |  | | This study |
| CH368 | ∆*lac::FRT galK::cfp-bla yfaH::FRT gyrA(S83L)* | 0.38 |  | | This study |
| CH369 | ∆*lac::FRT galK::cfp-bla yfaH::FRT gyrA(D87N)* | 0.25 |  | | This study |
| CH370 | ∆*lac::FRT galK::cfp-bla* ∆*marR::FRT* | 0.047 |  | | This study |
| CH371 | ∆*lac::FRT galK::cfp-bla* ∆acr*R::FRT* | 0.047 |  | | This study |
| CH372 | ∆*lac::FRT galK::yfp-bla* | 0.023 |  | | This study |
| CH373 | ∆*lac::FRT galK::yfp-bla yfaH::FRT gyrA(S83L)* | 0.38 |  | | This study |
| CH374 | ∆*lac::FRT galK::yfp-bla yfaH::FRT gyrA(D87N)* | 0.25 |  | | This study |
| CH375 | ∆*lac::FRT galK::yfp-bla* ∆*marR::FRT* | 0.047 |  | | This study |
| CH376 | ∆*lac::FRT galK::yfp-bla* ∆acr*R::FRT* | 0.047 |  | | This study |

**Table S2. Exponential growth rate data**

| **Sensitive strain (DA6192)** | | |  |  |  |  |  |
| --- | --- | --- | --- | --- | --- | --- | --- |
|  |  |  |  |  |  |  |  |
| Tetracycline conc (µg/ml) | Average growth rate  (h^-1^) | Average growth rate (min^-1^) | Relative growth rate | Stdev  (min^-1^) | Stdev (relative) | N | SEM (relative) |
| 0 | 1.72679 | 0.02878 | 1.00000 | 0.00071 | 0.02473 | 6 | 0.01010 |
| 0.0375 | 1.57588 | 0.02626 | 0.91261 | 0.00050 | 0.01733 | 8 | 0.00613 |
| 0.05 | 1.47459 | 0.02458 | 0.85395 | 0.00063 | 0.02198 | 14 | 0.00587 |
| 0.075 | 1.30768 | 0.02179 | 0.75729 | 0.00077 | 0.02666 | 12 | 0.00770 |
| 0.1 | 1.16295 | 0.01938 | 0.67347 | 0.00092 | 0.03182 | 13 | 0.00882 |
| 0.15 | 0.94863 | 0.01581 | 0.54936 | 0.00098 | 0.03394 | 14 | 0.00907 |
| 0.2 | 0.81904 | 0.01365 | 0.47432 | 0.00060 | 0.02087 | 14 | 0.00558 |
| 0.3 | 0.53817 | 0.00897 | 0.31166 | 0.00035 | 0.01232 | 14 | 0.00329 |
| 0.4 | 0.39358 | 0.00656 | 0.22793 | 0.00052 | 0.01812 | 13 | 0.00502 |
| 0.6 | 0.22666 | 0.00378 | 0.13126 | 0.00017 | 0.00588 | 14 | 0.00157 |
| 0.8 | 0.13916 | 0.00232 | 0.08059 | 0.00026 | 0.00910 | 14 | 0.00243 |
|  |  |  |  |  |  |  |  |
|  |  |  |  |  |  |  |  |
| **Resistant strain (DA17822)** | | |  |  |  |  |  |
|  |  |  |  |  |  |  |  |
| Tetracycline conc (µg/ml) | Average growth rate (h^-1^) | Average growth rate (min^-1^) | Relative growth rate | Stdev (min^-1^) | Stdev (relative) | N | SEM (relative) |
| 0 | 1.86071 | 0.03101 | 1.00000 | 0.00116 | 0.03734 | 7 | 0.01411 |
| 0.05 | 1.86183 | 0.03103 | 1.00060 | 0.00268 | 0.08637 | 8 | 0.03054 |
| 0.1 | 1.89517 | 0.03159 | 1.01852 | 0.00221 | 0.07110 | 7 | 0.02688 |
| 0.2 | 1.93856 | 0.03231 | 1.04184 | 0.00114 | 0.03678 | 8 | 0.01300 |
| 0.4 | 1.83837 | 0.03064 | 0.98799 | 0.00267 | 0.08599 | 8 | 0.03040 |
| 0.8 | 1.88495 | 0.03142 | 1.01303 | 0.00192 | 0.06183 | 8 | 0.02186 |
| 2 | 1.85582 | 0.03093 | 0.99737 | 0.00155 | 0.05002 | 8 | 0.01768 |
| 4 | 1.85509 | 0.03092 | 0.99698 | 0.00142 | 0.04574 | 8 | 0.01617 |
| 8 | 1.78401 | 0.02973 | 0.95878 | 0.00187 | 0.06035 | 8 | 0.02134 |
| 16 | 1.70180 | 0.02836 | 0.91459 | 0.00096 | 0.03081 | 8 | 0.01089 |
| 32 | 1.58493 | 0.02642 | 0.85179 | 0.00054 | 0.01735 | 8 | 0.00614 |
| 64 | 1.22447 | 0.02041 | 0.65806 | 0.00062 | 0.02012 | 8 | 0.00711 |
| 96 | 0.85867 | 0.01431 | 0.46147 | 0.00167 | 0.05376 | 8 | 0.01901 |
| 128 | 0.48747 | 0.00812 | 0.26198 | 0.00215 | 0.06937 | 6 | 0.02832 |

**Table S2**. Exponential growth rate data of a susceptible (DA6192) and a resistant (DA17822) strain of *S. typhimurium* when grown in single cultures at different tetracycline concentrations. Cells were grown in Mueller Hinton medium at 37°C with continuous shaking. OD_600_ measurements were made every 4 min and the calculations were based on OD_600_ values between 0.02 and 0.1, where growth was observed to be exponential.

**Table S3. Competition data.**

| **Selection coefficients streptomycin *rpsL105(K42R)* vs wild type** | | | | | |
| --- | --- | --- | --- | --- | --- |
| Conc. streptomycin (µg/ml) | 0 | 0.5 | 1 | 1.5 | 2 |
| Selection coefficient | -0.03197 | -0.01523 | -0.00154 |  | 0.04993 |
|  | -0.01412 | -0.01292 | -0.00010 | 0.00734 | 0.06256 |
|  | -0.02891 | -0.01076 | 0.00475 | 0.02893 | 0.05362 |
|  | -0.02209 |  | -0.00463 | 0.01551 | 0.06650 |
|  | -0.03027 | -0.01969 | -0.00858 | 0.01137 | 0.05113 |
|  | -0.02847 | -0.02162 | -0.00230 | 0.03149 | 0.07221 |
|  | -0.02804 | -0.02440 | 0.00050 | 0.03614 | 0.06213 |
|  | -0.02786 | -0.01001 | 0.00008 | 0.03754 | 0.06268 |
|  |  | -0.01911 | -0.01011 | 0.03061 | 0.06524 |
|  | -0.03173 | -0.02120 | 0.00306 | 0.03999 | 0.06231 |
|  |  | -0.01064 |  | 0.04240 | 0.08173 |
|  | -0.02815 | -0.02214 |  | 0.02688 | 0.06350 |
|  | -0.04267 | -0.03459 | -0.01293 |  | 0.04302 |
|  |  | -0.02637 | -0.01115 | 0.01059 | 0.06310 |
|  | -0.03617 | -0.02211 | -0.00438 | 0.03713 | 0.06107 |
|  |  | -0.02153 | -0.01521 | 0.03369 | 0.07180 |
|  | -0.01949 | -0.02316 | -0.01079 | 0.02040 | 0.06965 |
|  | -0.02766 |  | 0.00948 | 0.03117 | 0.07157 |
|  | -0.01670 | -0.01550 |  | 0.03336 | 0.06756 |
|  | -0.03626 |  | -0.00185 | 0.05735 | 0.07152 |
| Mean | -0.02816 | -0.01947 | -0.00387 | 0.02955 | 0.06364 |
| Stdev | 0.00736 | 0.00642 | 0.00678 | 0.01266 | 0.00900 |
| N | 16 | 17 | 17 | 18 | 20 |
| SEM | 0.00184 | 0.00156 | 0.00164 | 0.00298 | 0.00201 |
|  |  |  |  |  |  |
| **Selection coefficients tetracycline *cobA367*::Tn10*d*tet vs wild type** | | | | | |
| Conc. tetracycline (ng/ml) | 0 | 25 | 50 | 75 | 100 |
| Selection coefficient | -0.00072 | -0.00728 | 0.04040 | 0.10018 | *0.10343* |
|  | -0.00962 |  | 0.04352 | 0.08910 | *0.10102* |
|  | 0.00268 | 0.00637 |  | 0.09621 | *0.10773* |
|  | 0.00051 | 0.01372 | 0.04066 | 0.05229 | *0.10442* |
|  | -0.01460 | 0.02376 | 0.08785 |  |  |
|  | -0.01156 | 0.01999 | 0.05945 | 0.11517 |  |
|  | -0.02383 | 0.01237 |  |  |  |
|  | -0.00987 | 0.03103 | 0.05807 | 0.09175 |  |
|  | -0.00624 | 0.01596 | 0.05197 | 0.10134 | *0.12945* |
|  | -0.00996 | 0.01024 | 0.07016 | 0.09838 | *0.12405* |
|  | -0.01242 | 0.00130 | 0.03174 | 0.10334 | *0.13498* |
|  | -0.00730 | 0.01083 | 0.05309 | 0.09725 | *0.13782* |
|  | -0.01551 | 0.01807 | 0.05853 | 0.09298 | *0.13541* |
|  | -0.00812 | 0.00910 | 0.05775 | 0.08635 |  |
|  | -0.01941 | -0.00171 | 0.05781 | 0.09231 | *0.11041* |
|  | -0.01157 | -0.00953 | 0.01783 | 0.09189 | *0.13477* |
|  | -0.01384 | 0.00987 | 0.04443 |  |  |
|  | -0.02261 | 0.00106 | 0.04001 | 0.10557 | *0.09820* |
|  | -0.00989 | 0.00913 | 0.02929 | 0.08186 | *0.10229* |
| Mean | -0.01073 | 0.00968 | 0.04956 | 0.09350 |  |
| Stdev | 0.00702 | 0.01040 | 0.01648 | 0.01360 |  |
| N | 19 | 18 | 17 | 16 |  |
| SEM | 0.00161 | 0.00245 | 0.00400 | 0.00340 |  |

| **Selection coefficients ciprofloxacin *gyrA2(D87N)* vs wild type** | | | | | | | |
| --- | --- | --- | --- | --- | --- | --- | --- |
| Conc. ciprofloxacin (ng/ml) | 0 | 1.64 | 1.92 | | 2.3 | 2.88 | 3.83 |
| Selection coefficient | -0.03177 | -0.02245 | -0.00921 | | -0.00013 | 0.01450 | 0.04505 |
|  | -0.03090 | -0.01944 | -0.00646 | | -0.00086 | 0.01752 | 0.03877 |
|  | -0.02974 | -0.01615 | -0.00490 | | -0.00222 | 0.01125 | 0.04426 |
|  | -0.02169 | -0.00659 | -0.01902 | | -0.00679 | 0.01373 | 0.04699 |
|  | -0.02375 | -0.02766 | -0.01655 | | -0.00487 | 0.01261 | 0.06691 |
|  | -0.02663 | -0.02397 | -0.01013 | | -0.00692 | 0.00978 | 0.05803 |
| Mean | -0.02741 | -0.01938 | -0.01104 | | -0.00363 | 0.01323 | 0.05000 |
| Stdev | 0.00408 | 0.00739 | 0.00560 | | 0.00297 | 0.00270 | 0.01042 |
| N | 6 | 6 | 6 | | 6 | 6 | 6 |
| SEM | 0.00167 | 0.00302 | 0.00229 | | 0.00121 | 0.00110 | 0.00425 |
|  |  |  |  | |  |  |  |
| **Selection coefficients ciprofloxacin *gyrA1(S83L)* vs wild type** | | | | | | | |
| Conc. ciprofloxacin (ng/ml) | 0 | 0.23 | 0.46 | | 1.15 | 2.3 | 2.88 |
| Selection coefficient | 0.00003 | 0.00040 | 0.00061 | | 0.01323 | 0.05095 | 0.09622 |
|  | -0.01438 | 0.00438 | 0.00785 | | 0.02384 | 0.06254 | 0.09003 |
|  | 0.00563 | -0.00366 | 0.00087 | | 0.02187 | 0.06312 | 0.08381 |
|  | -0.00347 | 0.00415 | 0.01054 | | 0.03088 | 0.07884 | 0.09110 |
|  | -0.00067 | 0.00835 | 0.01116 | | 0.03066 | 0.07503 | 0.09368 |
|  | -0.00021 | 0.00387 | 0.01593 | | 0.02857 | 0.07625 | 0.10287 |
| Mean | -0.00218 | 0.00292 | 0.00783 | | 0.02484 | 0.06779 | 0.09295 |
| Stdev | 0.00667 | 0.00409 | 0.00607 | | 0.00677 | 0.01076 | 0.00640 |
| N | 6 | 6 | 6 | | 6 | 6 | 6 |
| SEM | 0.00272 | 0.00167 | 0.00248 | | 0.00276 | 0.00439 | 0.00261 |
|  |  |  |  | |  |  |  |
| **Selection coefficients ciprofloxacin *∆marR* vs wild type** | | | | | | | |
| Conc. ciprofloxacin (ng/ml) | 0 | 1.64 | 1.92 | | 2.3 | 2.88 | 3.83 |
| Selection coefficient | -0.03724 | -0.02138 | -0.00525 | | -0.00254 | 0.01530 | 0.03696 |
|  | -0.02283 | -0.01765 | -0.00734 | | -0.00322 | 0.00925 | 0.04285 |
|  | -0.02481 | -0.01841 | -0.00749 | | -0.00534 | 0.00870 | 0.03821 |
|  | -0.02500 | -0.01832 | -0.00101 | | -0.00032 | 0.01326 | 0.04675 |
|  | -0.02197 | -0.01664 | -0.00847 | | -0.00013 | 0.02077 | 0.04897 |
|  | -0.02176 | -0.01419 | -0.00427 | | 0.00159 | 0.01593 | 0.05275 |
| Mean | -0.02560 | -0.01776 | -0.00564 | | -0.00166 | 0.01387 | 0.04442 |
| Stdev | 0.00587 | 0.00236 | 0.00275 | | 0.00251 | 0.00452 | 0.00620 |
| N | 6 | 6 | 6 | | 6 | 6 | 6 |
| SEM | 0.00240 | 0.00096 | 0.00112 | | 0.00102 | 0.00185 | 0.00253 |
|  |  |  |  | |  |  |  |
| **Selection coefficients ciprofloxacin *∆acrR* vs wild type** | | | | | | | |
| Conc. ciprofloxacin (ng/ml) | 0 | 1.64 | 1.92 | 2.3 | | 2.88 | 3.83 |
| Selection coefficient | -0.02241 | -0.00301 | 0.00215 | 0.01192 | | 0.02481 | 0.06024 |
|  | -0.01467 | -0.00414 | 0.00064 | 0.01261 | | 0.03821 | 0.05265 |
|  | -0.01586 | -0.00404 | 0.00293 | 0.01370 | | 0.02992 | 0.05657 |
|  | -0.02912 | -0.01906 | -0.01179 | -0.00956 | | 0.00569 | 0.03760 |
|  | -0.02599 | -0.02291 | -0.01482 | -0.00855 | | 0.00830 | 0.04613 |
|  | -0.02660 | -0.01692 | -0.01576 | -0.00881 | | 0.00491 | 0.03394 |
| Mean | -0.02244 | -0.01168 | -0.00611 | 0.00189 | | 0.01864 | 0.04785 |
| Stdev | 0.00597 | 0.00893 | 0.00891 | 0.01191 | | 0.01422 | 0.01053 |
| N | 6 | 6 | 6 | 6 | | 6 | 6 |
| SEM | 0.00244 | 0.00364 | 0.00364 | 0.00486 | | 0.00581 | 0.00430 |

| **Selection coefficients tetracycline *cobA367::*Tn*10d*Tet vs wild type** | | | | |
| --- | --- | --- | --- | --- |
|  |  |  |  |  |
| Conc. tetracycline | 0 | 30 | 60 | Initial ratio S:R |
| Selection coefficient | -0.01288 | 0.03213 | 0.07291 | 10:1 |
|  | -0.00252 | 0.01594 | 0.07362 |  |
|  | -0.00347 | 0.01754 | 0.07148 |  |
|  | -0.00965 | 0.01970 | 0.07205 |  |
|  | -0.00943 | 0.03185 | 0.07185 |  |
|  | -0.00466 | 0.01754 | 0.06735 |  |
|  | 0.00559 | 0.01370 | 0.07655 | 10^2^:1 |
|  | -0.02128 | 0.02710 | 0.08530 |  |
|  | -0.01010 | 0.02115 | 0.07155 |  |
|  | -0.01050 | 0.01370 | 0.07379 |  |
|  | -0.02176 | 0.02365 | 0.07723 |  |
|  | -0.00314 | 0.01958 | 0.08135 |  |
|  |  | 0.01302 | 0.07797 | 10^3^:1 |
|  | -0.01776 | 0.01447 | 0.08107 |  |
|  | -0.01237 | 0.02122 | 0.07568 |  |
|  | -0.00830 | 0.02254 | 0.08088 |  |
|  | -0.00577 | 0.01722 | 0.07874 |  |
|  | -0.01059 | 0.01763 | 0.07867 |  |
|  | -0.01400 | 0.00691 | 0.08060 | 10^4^:1 |
|  | -0.00532 | 0.01525 | 0.08792 |  |
|  | -0.00257 | 0.02724 | 0.08073 |  |
|  | -0.00248 | 0.02062 | 0.08443 |  |
|  | 0.00229 | 0.02125 | 0.08067 |  |
|  | -0.00484 | 0.01945 | 0.06811 |  |
| Mean | -0.00807 | 0.01960 | 0.07710 |  |
| Stdev | 0.00681 | 0.00592 | 0.00538 |  |
| N | 23 | 24 | 24 |  |
| SEM | 0.00142 | 0.00121 | 0.00110 |  |

**Table S3**. Competition data, where the selection coefficients are calculated from the slopes of the ln ratio of resistant/susceptible strains as a function of the number of generations. Each s-value is calculated from one single competition. Competitions where contaminations or mutations have occurred (non linear slope) are excluded from the calculations.

**Appendix**

**Fixation time for an adaptive mutation**

Consider a bacterial population of constant effective size *N*. Mutations to resistance occurs with rate *u* per genome replication. In the absence of antibiotic, the resistance mutation has a selection coefficient *s*_0_ < 0 relative to wild type. In the presence of antibiotic, the selection coefficient is *s* (*s* > 0 assumed); see Fig. 2 and 3 of the main text. Assuming that antibiotic is added at time *t* = 0, what is the mean time before the resistance mutation has taken over 50% of the population?

Mutants will arise in the population with rate *uN*. With probability *s*/[1-exp(-*sN*)] ≈ *s*, a new mutant will survive and spread in the population. Thus, the mean time for the first surviving mutant to appear is 1/(*uNs*) if none were present initially. Thereafter its presence in the population will grow deterministically. The fraction of mutants at time *t* will change according to

$\frac{df}{dt}=u(1-f)+sf(1-f)$ (1)

Given a starting fraction *f*_0_ in the population, the solution to this equation is

$f(t)=\frac{f_{0}+\left(u/s\right)\left[1-\left(1-f_{0}\right)\exp\left(-\left(s+u\right)t\right)\right]}{f_{0}+\left(u/s\right)+\left(1-f_{0}\right)\exp\left(-\left(s+u\right)t\right)}$ (2)

The time *t* = *T*_50_ when *f*(*t*) = 0.5 is given by

$T_{50}=\frac{1}{s+u}\ln\left(\frac{\left(1+2u/s\right)\left(1-f_{0}\right)}{f_{0}+u/s}\right)\approx\frac{1}{s}\ln\left(\frac{1-f_{0}}{f_{0}+u/s}\right)$ (3)

The approximation holds in the reasonable limit where *u* << *s*.

$T_{50}^{0}=\frac{1}{uNs}+\frac{1}{s}\ln\left(\frac{sN}{uN+s}\right)$ (4)

For large population sizes such that *uN* > 1, mutants are likely to be present initially. If there are *m*_0_ mutants present initially, *f*_0_ = *m*_0_/*N* (<<1 assumed), the mean mutant penetration time is

$T_{50}^{in}=\frac{1}{s}\ln\left(\frac{sN}{uN+sm_{0}}\right)$ (5)

A large population (with *uN* > 1) that has reached mutation-selection balance before addition of antibiotics would be expected to have *m*_0_ = *Nf*_0_ = *uN*/|*s*_0_| mutants present initially. If the starting number of mutants is low, *m*_0_ < 10 or so, there is a significant probability, = (1-*s*)*^m^*^0^ ≈ exp(-*m*_0_*s*), that they will be lost before they can grow deterministically. If this happens, the system will have to wait for new surviving mutants to appear. Thus, the expectation time for 50% penetration will be given by

$T_{50}\left(m_{0}\right)=\mathrm{e}^{-m_{0}s}T_{50}^{0}+\left(1-\mathrm{e}^{-m_{0}s}\right)T_{50}^{in}$ (6)

We have tested these results using a more complete stochastic model, a Moran model with overlapping generations. Individuals carrying the mutation have a faster growth rate by a factor 1+*s*. The probability, *P_n_*(*t*), that the population contains *n* mutations at time *t* obeys the master equations

$\frac{dP_{n}}{dt}=k_{n-1}^{+}P_{n-1}-\left(k_{n}^{+}+k_{n}^{-}\right)P_{n}+k_{n+1}^{-}P_{n+1}; 0\leq n\leq N$ (7)

Adapting the model by Berg and Kurland[31] to the present situation, the rates of change can be expressed as

$\begin{matrix} k_{n}^{+}=\left[\left(1+s-u\right)n+uN\right]\frac{N-n}{N+1} \\ k_{n}^{-}=\frac{n\left(N-n\right)}{N+1} \text{for} n<N/2 \\ k_{n}^{-}=0 \text{for} n=N/2 \end{matrix}$ (8)

As we want to calculate the time to reach *n* = *N*/2, it is assumed that this step is irreversible such that $k_{n}^{-}=0$ for *n* = *N*/2. Then the first passage time can be calculated from the sum of the residence times in the states below *n* = *N*/2

$T_{50}=\sum_{n=0}^{N/2-1} \tau_{n}$ (9)

The residence time in each state is determined by $\tau_{n}=\int_{0}^{\infty} P_{n}(t)dt$. Assuming that the system starts in the state *n* = *m*_0_, and for simplicity of notation setting *m* = *N*/2 -1, one finds by integration of the master equations (Eq. 7) the following recursive relations for *t_n_*:

$\begin{matrix} \tau_{m}=\frac{1}{k_{m}^{+}} , \\ \tau_{m-j}=\frac{1}{k_{m-j}^{+}}\left(1+k_{m+1-j}^{-}\tau_{m+1-j}\right) \text{for} j=1,...m-m_{0} \\ \text{and} \\ \tau_{m-j}=\frac{k_{m+1-j}^{-}}{k_{m-j}^{+}}\tau_{m+1-j} \text{for} j=m+1-m_{0},...m \end{matrix}$ (10)

the mean first passage times calculated from the more intuitive Eq. (6) agree very well with the results from Eqs. (7)-(10), see Fig. 6.

The most serious situation in terms of fixation of a resistance mutation is when *uN* > 1. In this limit, a resistance mutation is likely to be present initially, but even if it were not, there is a very short waiting time to get the first one and *T*_50_ = (1/*s*)ln(*s*/*u*) holds reasonably well (cf. solid lines in Figs.6A and B). When *uN* < |*s*_0_|, it is less likely that any mutant is present initially and the expected penetration time is dominated by the waiting time 1/(*uNs*); cf. dash-dot line in Fig. 6A.
